# Global community perception of surgical care as a public health issue: a cross sectional survey

**DOI:** 10.1186/s12889-021-10936-0

**Published:** 2021-05-20

**Authors:** Nurhayati Lubis, Meena Nathan Cherian, Chinmayee Venkatraman, Fiemu E. Nwariaku

**Affiliations:** 1 Geneva Foundation for Medical Education and Research, Chemin de Beau-Soleil 12, 1206 Geneva, Switzerland; 2 Office of Global Health, University of Texas Southwestern Medical Centre, Dallas, TX USA

**Keywords:** Surgical care, Public health education, Universal health coverage, Sustainable development goals

## Abstract

**Background:**

In the last decade surgical care has been propelled into the public health domain with the establishment of a World Health Organisation (WHO) designated programme and key publications. The passing of the historic World Health Assembly Resolution (WHA) acknowledged surgical care as a vital component towards achieving Universal Health Coverage (UHC). We conducted the first worldwide survey to explore the perception of surgical care as a public health issue.

**Method:**

The anonymous, cross sectional survey targeted worldwide participants across a range of professional backgrounds, including non-medical using virtual snowball sampling method (in English) using Google Forms (Google Inc., Mountain View, CA, USA) from 20th February 2019 to 25th June 2019. The survey questions were designed to gauge awareness on Sustainable Development Goals (SDGs), UHC, WHO programmes and key publications on surgical care as well as perception of surgical care as a priority topic in public health.

**Results:**

The survey was completed by 1954 respondents from 118 countries. Respondents were least aware of surgical care as a teaching topic in public health courses (27%; *n*=526) and as a WHO programme (20%; *n*=384). 82% of respondents were aware of UHC (*n*=1599) and of this 72% (*n*=1152) agreed that surgical care fits within UHC. While 77% (*n*=1495) of respondents were aware of SDGs, only 19% (*n*=370) agreed that surgery was a priority to meet SDGs. 48% (*n*=941) rated surgical care as a cost-effective component of Primary Health Care. 88% (*n*=1712) respondents had not read the WHA Resolution on Strengthening emergency and essential surgical care and anaesthesia as a component of UHC.

**Conclusion:**

There is still a widespread gap in awareness on the importance of surgical care as a public health issue amongst our respondents. Surgical care was not seen as a priority to reach the SDGs, less visible as a WHO programme and not perceived as an important topic for public health courses.

**Supplementary Information:**

The online version contains supplementary material available at 10.1186/s12889-021-10936-0.

## Background

Within this decade, there has been a massive drive to bring surgical care (including anaesthesia) into the global public health domain. The World Health Organisation (WHO) established the Emergency and Essential Surgical Care (EESC) programme in 2004 with the goal of ensuring the safety and efficacy of clinical procedures in anaesthesia, surgery, orthopaedics, and obstetrics [1]. In 2005, the Global Initiative for Emergency and Essential Surgical Care (GIEESC) was formed as the first coordinated global effort of multidisciplinary stakeholders to meet the need for emergency, anaesthesia, and surgical care in primary healthcare facilities worldwide [2]. The positioning of surgical care and anaesthesia services in the context of universal health coverage (UHC) in the WHO Executive Board agenda for the first time in 2014 led to the passing of the historic World Health Assembly (WHA) Resolution 68.15 on Strengthening emergency and essential surgical care and anaesthesia as a component of UHC in 2015 [3]. Additionally, the publication of a volume dedicated to Essential Surgery in the World Bank Disease Control Priorities 3rd Edition [4] and the launch of the Lancet Commission on Global Surgery Report [5] in the same year, were celebrated as major milestones, particularly within the surgical community.

The 2015 Sustainable Development Goals (SDGs) consisted of 17 goals to be achieved by 2030 and was adopted by 193 countries [6]. This marked a shift in the international communitys approach to favouring a horizontal and intersectoral systems approach rather than a vertical disease-specific one [7]. Recognising the necessity of surgical care to achieve both UHC and SDGs, the WHO and Lancet Commission recommended the development of national surgical, obstetric and anaesthesia plans (NSOAPs) [8]. Embedding NSOAPs into existing National Health Sector Strategic Plans will require engaging diverse stakeholders within the Ministry of Health (MoH), and can contribute to the achievement of SDGs 1, 3, 5, 8, 9, 10, 16, and 17 [9].

For these landmark events to translate into action, strong, consistent advocacy and commitment from multi-stakeholders are vital to engaging communities, donors and policy makers [10]. The general public should demand their basic right to timely, safe and affordable surgical care; the economist should see strengthening healthcare system to allow safe surgical services as a cost-effective investment; the health ministries should address this challenge by establishing essential surgical and anaesthesia services as part of broad national programmes; and the policy-makers should be reminded of their commitment to achieve UHC and SDGs to improve quality essential surgical care services.

For effective advocacy there needs to be recognition of the vital role that surgical care plays in meeting the SDGs and towards achieving UHC. Despite the high-profile advancements mentioned [1,5], we believe that there persists a lack of awareness on this topic as a public health priority amongst healthcare and public health professionals and the general population, apart from a select few. Therefore, we conducted a survey to explore the general awareness and understand the views on essential surgical care as a public health issue, worldwide. To our knowledge, this is the first multi-country survey to seek understanding of the perceived role of surgical care towards achieving UHC. Previous studies have been single-centred and limited to awareness of SDGs amongst university students and staff [11], UHC knowledge amongst nurses [12] and medical students perception of surgical care in context of global health [13].

## Method

The study protocol was cleared through the Institutional Review Board of the University of Texas Southwestern Medical Centre, which granted the study exempt status. We aimed to sample a diverse, cross-sectional global population. The study aimed to gauge public opinion surrounding SDGs, UHC, WHO programmes, and key documents relating to surgical care. To prevent influencing respondents, we titled the study tool Public Health Survey without mentioning surgical care in the title.

The survey questions were drafted by MNC and the draft revised by the other 3 authors to ensure face validity. The survey consisted of 14 questions (AdditionalFile1), mostly closed-ended format with checkboxes for ease of analysis. The last question gave respondents the opportunity to provide an open-ended comment. The first four questions were related to demographics; we aimed to include respondents from all six WHO regions [14] and a wide range of professional backgrounds, including non-medical. Respondents were disaggregated into the following categories: doctors, nurses, allied healthcare professionals, public health professionals, non-governmental organisations (NGOs), MoHs, university students, and other. We looked through the entries under other to see if any fit into our operational definitions of the 8 categories and corrected the data.

Questions 6 to 13 were designed to gauge the respondents knowledge and opinion on SDGs, UHC, WHO programmes, and key documents relating to surgical care. The wording and order of the questions was such that the respondents were not led to any specific answer, and no undefined abbreviations were used.

The survey was anonymous, and participation was voluntary. We did not collect respondents personal data apart from anonymous demographics. The survey took approximately 3minutes to answer, employed clear, simple English language, and was piloted on 20 volunteers with varying backgrounds in healthcare. Their feedback was taken into account and changes made to ensure the questions were clearly understood. The response from this pilot group was not included in the final analysis.

The survey was conducted over a 4-month period from 20th February 2019 to 25th June 2019. We did not set an aim for the number of respondents, as the study was not powered, and no statistical significance analysis was carried out. The survey was completed online using Google Forms (Google Inc., Mountain View, CA, USA) with a user-friendly interface. The survey link was shared via email networks to a wide range of recipients. The recipients were encouraged to further share the survey widely through their global networks to achieve a virtual snowball sampling method [15]. To increase coverage, the survey link was also circulated via social media platforms including WhatsApp, Twitter, and Facebook.

Google Forms software automatically collated the study data by generating an Excel worksheet which was used for data analysis. We used relative frequency statistics to analyse the closed questions. For the free text comments, the responses were organised into themes and formed the basis of an interpretative discussion.

The questionnaire was designed such that respondents were not able to progress to the next question without choosing an answer option using checkboxes. The only parts that were non-mandatory were the two free text sections (country of origin and comments). Seven respondents did not disclose their country of origin, but we did not exclude them as they answered all the mandatory questions.

## Results

The survey was completed by a total of 1954 respondents from 118 countries across all six WHO regions. Table1 summarises the respondents by WHO regions; African (28%; *n*=550), the Americas (26%; *n*=506), South Eastern Asia (25%; *n*=491), European (10%; *n*=186), Eastern Mediterranean (7%; *n*=134) and Western Pacific (4%; *n*=80). Seven respondents did not state their country of origin.

**Table 1:** Respondents by WHO regions

European		The Americas		African		Eastern Mediterranean		South Eastern Asia		Western Pacific	
Belgium	1	Argentina	2	Angola	1	Afghanistan	12	Bangladesh	7	Australia	27
Bulgaria	1	Bahamas	1	Burkina Faso	15	Bahrain	2	Bhutan	118	Cambodia	2
Bosnia	1	Belize	1	Burundi	6	Egypt	7	Thailand	21	Fiji	3
Croatia	8	Bolivia	2	Botswana	2	Iran	14	India	271	China/Taiwan	4
Czech Republic	1	Canada	36	Cameroon	13	Iraq	4	Indonesia	41	Japan	14
Denmark	3	Colombia	3	Chad	1	Kuwait	3	Myanmar	7	Laos	3
France	7	Chile	2	DRC	6	Jordan	4	Nepal	25	Malaysia	5
Georgia	1	Brazil	7	South Sudan	7	Lebanon	1	Sri Lanka	1	Mongolia	5
Germany	19	Guatemala	1	Ethiopia	95	Morocco	2	**Total**	**491**	New Zealand	3
Greece	1	Haiti	2	Gambia	3	Pakistan	31			PNG	1
Hungary	1	Honduras	11	Ghana	28	Saudi Arabia	3			South Korea	3
Italy	4	Mexico	72	Guinea	1	Somalia/Somaliland	12			Singapore	3
Luxemburg	1	Nicaragua	4	Ivory Coast	2	Syria	1			Vietnam	5
Malta	1	Paraguay	4	Lesotho	1	Tunisia	1			Philippines	2
Macedonia	1	Peru	5	Liberia	2	Yemen	4			**Total**	**80**
Netherlands	8	St Kitts	1	Malawi	7	UAE	4				
Romania	1	Trinidad	1	Mozambique	3	Sudan	22				
Norway	3	USA	350	Mali	3	Eritrea	1				
Portugal	1	Uruguay	1	Namibia	4	Palestine	6				
Poland	2	**Total**	**506**	Niger	1	**Total**	**134**				
Spain	1			Nigeria	140						
Switzerland	16			Rwanda	6						
Turkey	8			Senegal	1						
Uzbekistan	1			Sierra Leone	20						
Ukraine	1			South Africa	6						
United Kingdom	93			Swaziland	1						
**Total**	**186**			Tanzania	7						
				Uganda	66						
				Zambia	59						
				Zimbabwe	8						
				Kenya	35						
				**Total**	**550**						

The sex distribution of respondents was fairly equal with 51% male (*n*=995) and 49% (*n*=959) female. The age distribution was more widespread with the greatest proportion of respondents belonging to the 26 to 35years (28%, *n*=556), followed by 25% between 36 to 45years (*n*=492). Only 8% were over 66years (*n*=156).

**Fig. 1:**
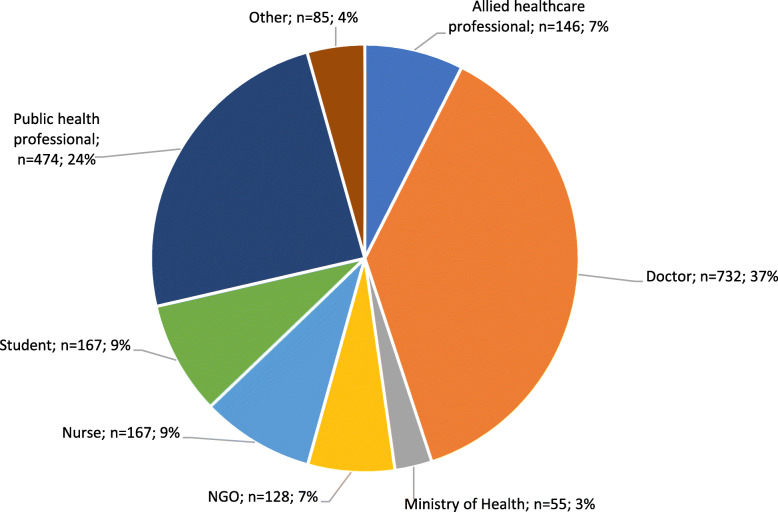
Professional Affiliation

The professional affiliation of the respondents is shown in Fig.1. The largest proportion of respondents were doctors at 37% (*n*=732), followed by public health professionals at 24% (*n*=474) and nurses at 9% (*n*=167). Of the 9% in the student subgroup, the majority were medical students (77% *n*=129).

**Fig. 2:**
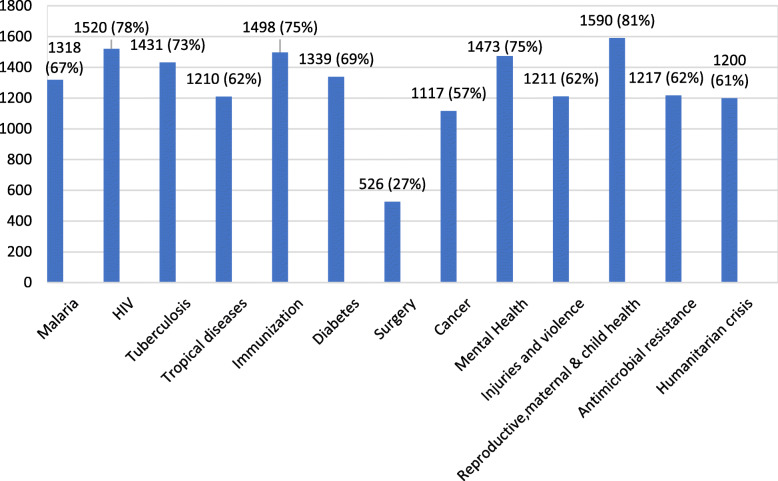
Which of the following topics should be taught in public health courses?

When asked what topics should be taught in public health courses (Fig.2), reproductive, maternal and child health ranked highest at 81% (*n*=1590). Human Immunodeficiency Viruses (HIV), tuberculosis (TB), immunisation, and mental health also ranked high (over 70%). All the other topics (antibiotic resistance, diabetes, injuries and violence, malaria and humanitarian crisis) scored over 60%, with the exception of cancer at 57% while surgery lags far behind with only 27% (*n*=526).

**Fig. 3:**
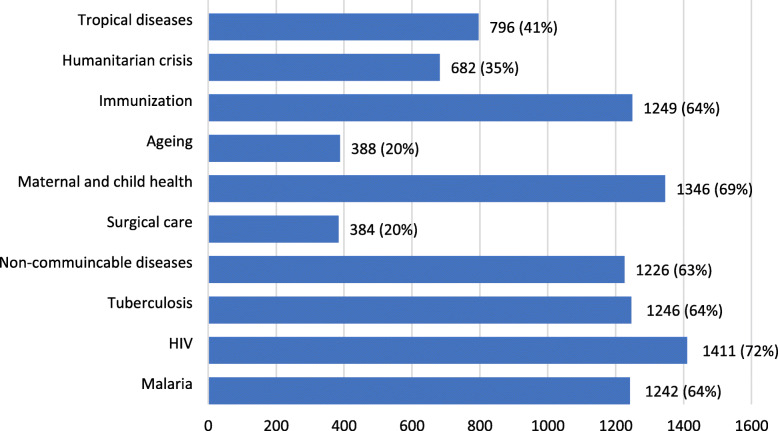
Which of the WHO programmes are you aware of?

On evaluating respondents knowledge of WHO programmes, Fig.3 shows that over 60% of respondents identified programmes in HIV, maternal and child health, TB, malaria, immunisation and non-communicable diseases (NCDs). Respondents were less familiar with programmes in tropical diseases (41%; *n*=796) and humanitarian crisis (35%; *n*=682). Even fewer respondents acknowledged surgical care as a WHO programme at 20% (*n*=384), which was on par with ageing (*n*=388).

**Fig. 4:**
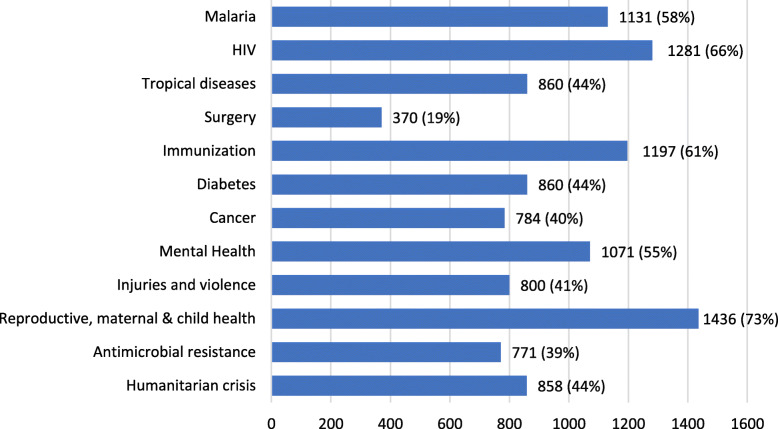
Which of the following health issues are a priority to reach the SDGs?

With respect to international health policy, 82% (*n*=1599) respondents were aware of UHC. Of this, 72% (*n*=1152) agreed that surgical care fits within UHC, while 14% (*n*=220) said it did not and 14% did not know. In comparison, 77% respondents were aware of SDGs (*n*=1495) but only 19% (*n*=370) think that surgery is a priority to reach SDGs, which again is by far the lowest compared to 73% (*n*=1436) agreeing with reproductive, maternal and child health as a priority (Fig.4).

Only 25% of the respondents (*n*=494) left a comment in the free text box provided at the end of the survey. However, after excluding unrelated comments (thank you, no comments, or email addresses) the number dropped to 394 and relevant comments are further considered.

Less than half respondents (48%; *n*=941) rated surgical care as a cost-effective component of Primary Health Care while 28% (*n*=549) think surgical care is not and the remainder (24%; *n*=464) did not know. This was also reflected in the comments section where respondents voice their opinion on the unaffordability of providing surgical care at personal level due to out of pocket expenditure and also at the governmental level (*n*=13). Comments include surgical care is a last resort intervention, a very expensive operation that most people cant afford and most public health specialists dont focus on surgical procedures as we rather focus on solving public health issues in a cost-effective wa*y*.

**Fig. 5:**
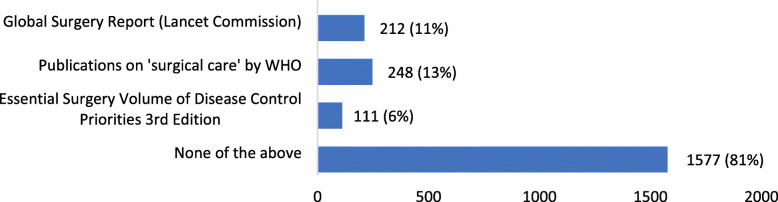
Which of the publications have you read?

The overwhelming majority of 88% (*n*=1712) respondents had not read the WHA Resolution on surgical care and anaesthesia; and 81% (*n*=1577) had not read any of the other three publications (Essential Surgery Volume of Disease Control Priorities, 3rd edition; Global Surgery Report by the Lancet Commission; Publications on surgical care by WHO) as summarised in Fig.5. In their comments, 28 respondents asked for access to these documents.

## Discussion

It is encouraging to see that the majority of respondents were aware of both UHC and SDGs (82 and 77% respectively). This is an improvement from previous studies [11,13]. Although those who were aware of UHC in our survey vastly agree that surgical care fits within UHC, only 19% agree that surgical care is a priority to reach the SDGs. Furthermore, only 20% were aware of the WHO programme on surgical care and only 27% of respondents think surgical care has a place in public health education. There is a disconnect between the respondents awareness of UHC and SDGs and recognition of surgical care as a public health topic towards achieving this. Surgical care is still not as visible as other public health topics and remains an outsider.

Several public health programmes including maternal and child health, NCDs, humanitarian crisis and tropical diseases clearly involves access to essential surgical services [16]. Still, these programmes often seem to be implemented exclusive of strengthening surgical care services and understood better as priority public health issues as reflected in our survey (Fig. 3). Perhaps this is attributed to the fact that surgical care often does not fit into a vertical programme which is easier to implement with easily measurable endpoints [10]. Surgical care needs to be promoted to all stakeholders as an important cross-cutting essential service for improving quality and safety of patient care from primary to tertiary level healthcare.

Access to surgical services when required, is a crucial component across diseases and throughout our lives. Lack of timely and affordable access to surgery increases the burden of diseases [4], affects economic productivity [17] and psychosocial health [18]. While surgical care is entwined across most public health issues, surgical care alone was not seen as a significant topic by our respondents even though the majority were aware of the UHC and SDGs. Surgery will continue to lag behind in support unless we increase public appreciation on the role of surgical care services in UHC. The Lancet Commission on Global Surgery group recognises the importance of active, prospective engagement strategy by generating a dedicated website and via social media [19]. Through emphasis on education and advocacy, surgical care could be seen on par with other public health initiatives allowing integration in policies and planning at the national level.

It is recognised that surgical care is critical for primary health care and UHC [2, 3]. Promisingly, 48% of our respondents agree that surgical care is a cost-effective component of Primary Health Care. There is still a false belief amongst the general public that surgical care is expensive, accepting it as a luxury for the fortunate few in low and middle income countries (LMICs) [20, 21]. Policy makers faced with cost-constraints may wrongly overlook surgical provision as something unachievable and less cost-effective than other health interventions. This lack of understanding is yet another barrier. Although the financial cost of surgical expansion is significant, the cost of inaction on national incomes is much greater [5, 22]. Surgical diseases accounted for more than 15% of the total disability-adjusted life years (DALYs) lost worldwide, which is more than HIV, TB, and malaria combined [23]. Unfortunately, these strong messages have not been promoted in a simple manner to reach the grassroots, service users and the stakeholders. From our survey it is clear that HIV, TB and malaria remains the favoured topics for public health education in comparison to surgical care (Fig. 2).

The term surgical care itself was seen as being too broad and there was confusion amongst our respondents over what procedures fall under surgical care as stated in their comments (*n*=5). Several attempts have been made to address this [24, 25] and the term surgical conditions is often used in parallel with surgical disease although neither is consistently defined in literature [20]. This reflects the widespread unfamiliarity on this topic. The World Bank Disease Control Priorities 3rd Edition on Essential Surgery identifies 44 surgical procedures as essential on the basis that they address substantial needs, are cost effective, and are feasible to implement [4]. The Bellwether Procedures (caesarean delivery, laparotomy and treatment of open fracture) was introduced as a benchmark for what first level hospitals should be delivering in order to provide emergency and essential surgical care to their population [26]. Having a well-established, clear common definition is central to the setting of objectives, priorities, and strategies, communication of goals, and directing of resources [27]. Misperception over the definition could lead to fractions across groups instead of cohesion and cooperation as seen in the global health context [28].

Less than 20% of respondents have read one or more of the key publications specific to surgical care which are freely available online. We concur that these publications make for a lengthy, concentrated read, thus may not appeal to the public in general. Twenty-eight respondents asked for access to these documents, showing there is lack of awareness not only on the existence of these publications but also the availability to freely download them. The request for more information by our survey respondents was encouraging, and we should take advantage of this to promote and achieve surgical care as a public health topic for all including utilising distance learning and incorporating it into public health curriculum. Indeed, 29 of our respondents in their comments welcomed more training, workshops and online courses for surgical care topic in public health. The positive impact of wider access of actionable health information has already been shown [29] and we should use this example to do more to help disseminate the information from the publications [3,5] in a simplified, and freely accessible manner such as via factsheets or toolkits [30], visual imagery [31] or public health education courses. We need to learn from other successful public health advocacy programmes [32, 33] in order to increase the visibility of surgical care.

We acknowledge some limitations to our survey. As we were relying on virtual snowball sampling method, we have no control on the survey sample population or size and the response rate is unknown. We could not follow up non-responders. Furthermore, the survey was only in English language and only accessible online. However, the snowball sampling method has been shown to be purposeful method of data collection in qualitative research [34]. We kept the title of our survey generic (public health survey) to avoid demand characteristics bias or selection bias if potential responders think the topic was not relevant to them or that they were not knowledgeable enough to participate in a survey specifically related to surgical care. Some comments (*n*=13) did point out that the survey may be biased towards surgical care. Indeed, the intention of our survey was to gauge the general understanding of surgical care as a part of the public health agenda towards achieving UHC through the SDGs.

Despite these limitations, the survey attracted a large number of respondents across a wide range of professions globally with a good spread across high income countries (HICs) and low and middle income countries (LMICs). The sex distribution was almost equal, and the respondents were mainly of working age group. We felt this demographic met our broad target population and resulted in a meaningful outcome. However, we acknowledge that our findings cannot be generalised. Further studies should target a more specific population and utilise a method that can be better controlled against bias within the dataset.

## Conclusion

We have conducted the first worldwide survey to seek understanding of the perceived role of surgical care towards achieving UHC. Although the passing of the historic WHA resolution by the WHO 194 member states was celebrated as key achievement especially by the surgical community, we have shown that a large gap in awareness exists on the importance of surgical care as a public health issue amongst our global respondents. Surgical care is not seen as a priority to reach the SDGs, less visible as a WHO programme and not perceived as an important topic for public health courses.

## Supplementary Information

**Additional file 1.**

## Data Availability

The datasets used and/or analyzed during the current study are available from the corresponding author on reasonable request.

## Abbreviations

WHO: World health organisation
EESC: Emergency and essential surgical care
GIEESC: Global initiative for emergency and essential surgical care
UHC: Universal health coverage
WHA: World health assembly
SDGs: Sustainable development goals
NSOAPs: National surgical, obstetrics and anaesthesia plans
MoH: Ministry of health
NGOs: Non-governmental organisations
HIV: Human immunodeficiency viruses
TB: Tuberculosis
NCDs: Non-communicable diseases
DALYs: Disability-adjusted Life years
LMICs: Low and middle income countries
HICs: High income countries
