# Supplementary material for: Global community perception of surgical care as a public health issue: a cross sectional survey
Source: BMC Public Health. 2021 May 20;21:958. doi: 10.1186/s12889-021-10936-0 (PMC8139156; doi:10.1186/s12889-021-10936-0)
Supplement: Supplementary file 1 — Additional file 1. [file 12889_2021_10936_MOESM1_ESM.docx]

**Global community perception of** **‘surgical care’ as a public health issue: a cross sectional survey**

Nurhayati Lubis^1*^, Meena Nathan Cherian^1^, Chinmayee Venkatraman^2^, Fiemu E. Nwariaku^2^

^1^Geneva Foundation Medical Education and Research, Geneva, Switzerland.

^2^ Office of Global Health, University of Texas Southwestern Medical Centre, Dallas, Texas, United States of America.

*Corresponding author: Nurhayati Lubis ([nlubis@doctors.org.uk](mailto:nlubis@doctors.org.uk)); Geneva Foundation for Medical Education and Research, Emergency and Surgical Care , 150, route de Ferney,

1211 Geneva 2 - Switzerland

**Supplement 1**

**Survey: Public Health**

(This is for printing and available as online link on GFMER website for survey participants)

*We at the Geneva Foundation for Medical Education and Research are interested in understanding people’s awareness of public health issues to develop public health training courses on topics of specific interests, and would greatly appreciate if you could give* ***3 minutes*** *of your time to complete our survey. This survey is anonymous and your participation is voluntary. Thank you.*

1. **Gender:**
2. Male
3. Female
4. **Age in years:**
5. 17-25
6. 26-35
7. 36-45
8. 46-55
9. 56-65
10. 66 and over
11. **Country:**
12. **Which of the following closely matches your profile or affiliation** (check one)**?**
13. Medical student
14. Nursing student
15. Nurse
16. Allied Healthcare professional
17. Doctor
18. Public health professional
19. NGO
20. Ministry of Health
21. Other, please specify:
22. **Which of the following topics should be taught in public health courses** (check all that apply)**?**
23. Malaria
24. HIV
25. Tuberculosis
26. Tropical diseases
27. Immunization
28. Diabetes
29. Surgery
30. Cancer
31. Mental health
32. Injuries and Violence
33. Reproductive, maternal and child health
34. Antimicrobial resistance
35. Humanitarian crisis
36. **Are you aware of the Sustainable Development Goals (SDGs)?**

Yes /No

1. **Which of the following health issues are a priority to reach the SDGs** (check all that apply)**?**
2. Malaria
3. HIV
4. Tropical diseases
5. Surgery
6. Immunization
7. Diabetes
8. Cancer
9. Mental health
10. Injuries and Violence
11. Reproductive, maternal and child health
12. Antimicrobial resistance
13. Humanitarian crisis
14. **Do you know what is meant by "Universal Health Coverage" (UHC)?**

Yes /No

1. **In your opinion does ‘surgical care’ fit within the UHC?**

Yes /No/Do not know

1. **Which of the World Health Organization programs are you aware of** (check all that apply)**?**
2. Malaria
3. HIV
4. Tuberculosis
5. Non-Communicable disease (Diabetes, Cancer, Blindness, Deafness, Oral Health, Mental health, Violence and Injuries)
6. Surgical care
7. Maternal and child health
8. Ageing
9. Immunization
10. Humanitarian crisis
11. Tropical Diseases
12. **In your opinion is surgical care a cost-effective component of Primary Health Care?**

Yes/ No/ Do not know

1. **Have you read the World Health Assembly Resolution on surgical care and anaesthesia?**

Yes /No

1. **Which of the following publications you have read** (check all that apply)**?**
2. Global Surgery Report by the Lancet Commission
3. Essential Surgery Volume of Disease Control Priorities, 3^rd^ edition
4. Publications on ‘surgical care’ by the World Health Organization
5. None of the above
6. **Optional.** Please leave additional comments below:

**Thank you very much for completing the survey.**
